# Radical Treatment of Haloacetonitriles in Aqueous Systems: A Kinetic Study

**DOI:** 10.1021/acsestwater.5c00134

**Published:** 2025-05-13

**Authors:** Stephen P. Mezyk, Maya H. Rogalski, Anh N. Dang, David M. Bartels, D. Ransom Hardison, William J. Cooper

**Affiliations:** 1 Department of Chemistry and Biochemistry, California State University at Long Beach, 1250 N. Bellflower Blvd., Long Beach, California 90840, United States; 2 Radiation Research Laboratory, University of Notre Dame, Notre Dame, Indiana 46556, United States; 3 Beaufort Laboratory, National Ocean Service, National Oceanic and Atmospheric Administration, 101 Pivers Island Road, Beaufort, North Carolina 28516, United States; 4 Department of Environmental Engineer Sciences, Engineering School of Sustainable Infrastructure and Environment, Herbert Wertheim College of Engineering, University of Florida, 365 Weil Hall, Gainesville, Florida 32611, United States

**Keywords:** haloacetonitriles, advanced oxidation/reduction processes, kinetics, radical chemistry, rate constants

## Abstract

Haloacetonitriles (HANs) are important drinking water disinfection byproducts formed through the chlorination and chloramination of amino acids. Although HAN concentrations in treated water are usually lower than trihalomethanes, they are still of major concern due to their higher cyto- and genotoxicity. HANs undergo chemical transformations by hydrolysis on the hour to week time scales; however, for possible direct water reuse situations, their active removal using advanced oxidation/reduction processes (AO/RPs) may be required. We report here our systematic kinetic study of the four major AO/RP radiolysis species, oxidizing hydroxyl (·OH) and sulfate (SO_4_^–·^) radicals and reducing hydrated electron (e_aq_^–^) and hydrogen atoms (H^·^) with five HANs (mono-, di-, and trichloroacetonitriles and mono- and dibromoacetonitriles) in water measured using electron pulse radiolysis techniques. At ambient temperatures and pH 1–7, significant reactivity was found for e_aq_^–^ (*k* = (1–5) × 10^10^ M^–1^ s^–1^) and H^·^ atoms (*k* = (1 – 40 × 10^7^ M^–1^ s^–1^), but only minimal oxidation by ^·^OH (*k* = (0.6–10) × 10^7^ M^–1^ s^–1^) and SO_4_^–·^ (*k* = (0.2–4) × 10^6^ M^–1^ s^–1^) occurred. These data suggest that the large-scale AO/RP treatment of these contaminants will be effective for deaerated reducing systems, where the reductive electron-induced degradation of HANs will occur.

## Graphical abstract

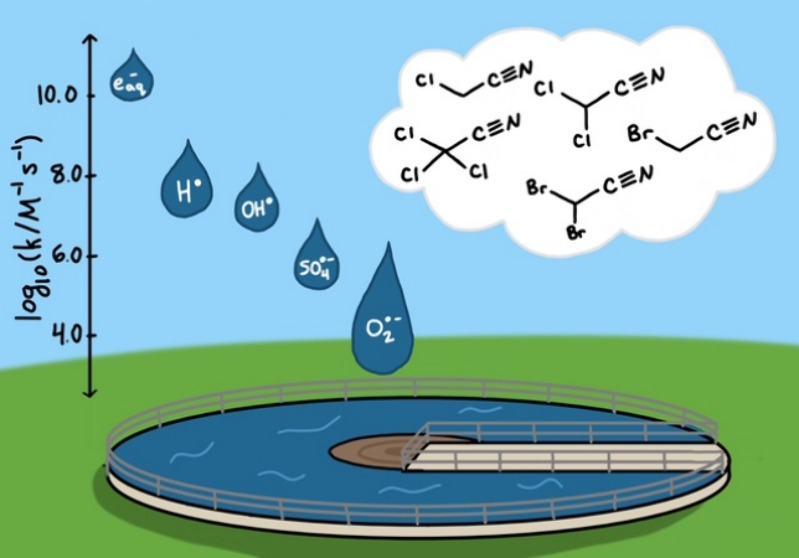

## Introduction

Thorough disinfection of drinking water is vital for maintaining public health.^1^ Chlorination has been used for this purpose since the early 1900s^2^ and has been effective at eliminating many prominent waterborne diseases, saving millions of lives.^3^ However, since the early 1970s concern has grown over this method of water treatment due to the formation of halogenated disinfection byproducts (DBPs). This is especially true in coastal cities where ocean salt intrusion may occur, creating high concentrations of bromide and subsequently brominated DBPs.^4–6^ Trihalomethanes (THMs) were first discovered in drinking water in 1974 ^7^ and maximum contaminant levels were established by the Environmental Protection Agency (EPA) in 1979.^8^ Examples of the major, problematic, DBPs formed as a result of chlorination include the trihalomethanes (THMs), haloacetic acids (HAAs), haloacetonitriles (HAN), halopicrin, cyanogen chloride and bromide, and chloral hydrate.^1,8^ Many of these DBPs are probable human carcinogens and mutagens,^11^ making their prescence in drinking water a major public health concern.

Disinfection byproduct control remains an important area of research due to the increasingly stringent EPA regulations for water treatment. There are many methods to treat drinking water, including use of chlorine, chloramines, chlorine dioxide, and ozone.^9^ However, all these methods result in the formation of DBPs from their reactions with ubiquitous natural organic matter (NOM). DBP formation is affected by many factors including temperature, concentration of NOM, and disinfection type.^10^ As such, the active removal of DBPs from treated waters may be required, particularly for direct reuse applications. Proposed treatment methods include water preozonation,^11^ UV–H_2_O_2_/persulfate photolysis,^12^ nanomaterial catalysts,^13,14^ and NOM removal.^15^ Adsorption techniques have also been demonstrated to have up to 90% efficiency for DBP elimination from water;^10,16^ however, some nitrogeneous-based DBPs are particularly toxic^17^ and may therefore require more quantitative removal.

Of particular concern are the HAN DBPs.^18^ HANs were first reported in drinking water in the 1980s,^19^ but originally escaped analysis due to their hydrolysis at higher pH**^20^** and their instability in solution after sample collection when reducing compounds had been added.^19^ The source of the HAN DBPs was initially thought to be fulvic acid and algae in water sources,^21^ but later studies showed that amino acids, either free or associated with NOM, were determined to be responsible for their formation.^22^ Where chlorination was used as the disinfectant, their concentrations were about 10% (on a molar basis) of the trihalomethane concentrations.^21,23–25^ When chlorine dioxide was the disinfectant, haloacetonitriles were only ∼ 3% of the trihalomethane concentration on a weight basis.^26^ However, multiple studies^27–29^ of these DBPs^30,31^ have reported that they have more significant adverse health effects compare to carbon-based DBPs, as based on Chinese hamster ovary cell assays.^27,32,33^

Studies of the environmental fate of HAN in wastewater effluent concluded that hydrolysis was their major degradation pathways.^34^ However, the rates of hydrolysis were extremely pH-dependent, ranging from hours (trihaloacetonitriles) to many days (chloroacetonitrile).^20^ This relatively slow degradation is of concern for water reuse plants, as although HAN levels are very low compared to the total organic halides generated in chlorinated waters, their higher toxicities^35^ suggest that they are the major contribution to the overall DBP-associated toxicity.^36^

Therefore, active removal of HANs and their degradation products by Advanced Oxidation/Reduction Processes (AO/RPs)^37^ is a novel remediation method of interest. These AO/RPs generate highly oxidizing and/or reducing radicals on demand in the treated water. Typically the oxidizing hydroxyl radical (^•^OH) is generated on a large scale through the use of UV/ozone or UV/peroxide combinations, photocatalysis of TiO_2_, or in an emerging application, ionizing radiation.^37^ These highly energetic ^•^OH (E° = 2.8 V vs. NHE^38^) radicals react with, and ultimately destroy chemical contaminants. More recently, the application of reducing AO/RP utilizing hydrated electron reactions, formed by either ionizing radiation or UV photolysis, has been proposed for water treatments for certain recalcitrant contaminants such as perfluorinated chemicals.^39^ However, the efficiency and cost of large-scale AO/RP remediation processes are critically dependent on many operating parameters, especially the reactivity of the generated radicals with the contaminants of concern.^40^ Thus, to establish the feasibility of using AO/RPs as a large-scale remediation technology for HANs the reaction kinetics of the radicals (or energetic species) with these contaminants in water need to be quantitatively established.

Unfortunately, to date very little data has been reported for the free radical reactions needed to evaluate the potential application of AO/RPs for HAN removal. One study examined the reaction of the oxidizing ^•^OH with dichloroacetonitrile; but no reaction rates were reported.^41^ Electron attachment has been studied in the gas phase over the temperature range of 295 – 556 K^42^ and reductive cleavage for haloacetonitriles (Cl, Br, I) has been reported in the aprotic solvent dimethylfuran.^43^ However, no comprehensive study of the absolute bimolecular reaction rate constants for the disinfection byproduct haloacetonitriles in water has yet been done. As a first step to examine the applicability of AO/RPs in water, wastewater and waters intended for reuse, in this work we have measured the absolute kinetic rate constant data for five important HANs, chloroacetonitrile, dichloroacetonitrile, trichloroacetonitrile, bromoacetonitrile and dibromoacetonitrile with the major oxidizing (hydroxyl, sulfate) and reducing (hydrated electron, hydrogen atom) radical species in water.

## Experimental Section

### Materials

The following chemicals were obtained and used at the highest purity available for the pulse radiolysis experiments; from Sigma-Aldrich Chemical Co.: bromoacetonitrile (97%), dibromoacetonitrile (97%), chlororacetonitrile (99%), dichloroacetonitrile (98+%), trichloroacetonitrile (98%), potassium thiocyanate (99%), potassium persulfate (99%) and sodium formate (ACS > 99%). Tertiary butanol (2-methyl-2-propanol) and methanol were sourced from Fisher Scientific, (ACS certified). Gases (Ar, N_2_, N_2_O and O_2_) were from Airgas at Ultrahigh purity. Aqueous solutions were made using deionized, Milli-Q quality (18.2 MΩ resistance, 13 μg L^–1^ TOC) water.

### Radiolysis studies

Electron pulse radiolysis was used for kinetic measurements as it allows for complete isolation of the specific reactions of interest with minimal solute addition. The irradiation of aqueous solution results in the formation of reactive free radicals and molecular products following the stoichiometry:^38,44^

$$H_{2}O\overset{radiolysis}{\to }{\left[0.28\right]}^{•}\mathrm{OH}+\left[0.27\right]{e^{-}}_{\mathrm{aq}}+\left[0.06\right]H^{•}+\left[0.07\right]H_{2}O_{2}+\left[0.27\right]H_{3}O^{+}+\left[0.05\right]H_{2}$$ 1

The numbers in brackets are the absolute yields of each species in units of μmol per Joule.^38^ Measurements of the reactivity of the strongly oxidizing hydroxyl (^•^OH) radical, the reducing aqueous electron (e^–^_aq_) and the sulfate radical (SO_4_^–•^, see later) were performed using the Model TB-8/16–1S linear electron accelerator at the Radiation Research Laboratory, University of Notre Dame, Indiana.^45,46^ This system produced 4–5 ns pulses of 8 MeV electrons to generate 1–3 μM radicals per pulse in a one-pass flow system. Solution flow was adjusted so that each pulse irradiated a fresh solution in the 1 cm flow cuvette. Transient absorption spectroscopy was used to follow these reactions with typically 10–20 pulses averaged to obtain a single kinetic trace. Dosimetry was based on the oxidation of thiocyanate ion (SCN^–^) to the thiocyanate radical dimer anion (SCN)_2_^–•^ for 10^–2^ M solutions in nitrous oxide saturated water.^47^

The Van de Graff accelerator at Argonne National Laboratory was used to determine the hydrogen atom (H^•^) rate constants.^48–50^ The method of choice for monitoring the change in hydrogen atom concentration following pulse radiolysis was direct electron paramagnetic resonance (EPR) as both the H^•^ and products only have relatively weak UV–visible absorption at short wavelengths. Rate constants of the hydrogen atom reaction were determined using the free-induction decay (FID) attenuation method because of the first-order kinetics obtained.^51,52^ Hydrogen atoms were generated in aqueous solutions within an EPR cavity using 5 ns pulses of 3 MeV electrons. Immediately after irradiation a 35 ns microwave pulse was applied to the sample resulting in free induction decay of the hydrogen atom low-field line (m_1_= 1/2). A LeCroy 7200 digital oscilloscope recorded the EPR transition with multiple pulses (500–2000) average to measure each FID at a repetition rate of 120 Hz.

Stock solutions were prepared by addition of HClO_4_ (0.10 M, Mallinckrodt A. R. Grade, 69.05%) and methanol (10^–2^ M) to Millipore-filtered water. Known volumes of this solution were added to the recirculating system, and then deoxygenated using argon. Exact acid concentrations were determined by calculation from standardization of the concentrated acid against 1.029 N HCl (Aldrich, volumetric standard). The solution was flowed through a flat cell in the cavity at a rate sufficient to ensure that each cell volume was completely replaced between pulses. The actual volume irradiated in each pulse was less than 0.10 mL Scavenging experiments were performed by successive injections of concentrated, solutes to the pH-adjusted stock water. The accuracy of these concentrations is estimated at better than 2%.

## Results and Discussion

### Hydrated Electron (e^–^_aq_) Rate Constants

The reduction kinetics for each individual HAN compound was determined by dissolving them into 500 mL of water containing 0.50 M *tert*-butanol that had been pre-saturated with Ar or N_2_ for at least 1 h at natural pH (ca. 6.8). The presence of this alcohol quickly converted the radiolytically formed hydroxyl radicals and hydrogen atoms into the relatively inert *tert*-butanol radical,^38^ according to

OH•+(CH3)3COH→H2O+CH2•(CH3)2COH⁣k2=6.6×108M−1s−1 2

H•+(CH3)3COH→H2+CH2•(CH3)2COH⁣k3=1.8×105M−1s−1 3

The isolated hydrated electron has a strong peak absorption at 720 nm (ε_720_ = 1.9 × 10^4^ M^–1^cm^–1 ^) in the visible-near IR region.^53^ The hydrated electron lives for several microseconds in pure water with its rate of disappearance increasing as the concentration of added HAN increases, as shown in Figure 1 for chloroacetonitrile (CAN). The reactions of the five HANs of interest with the hydrated electron did not produce any significant transient absorption in the UV–visible region (270–800 nm).

**Figure 1:**
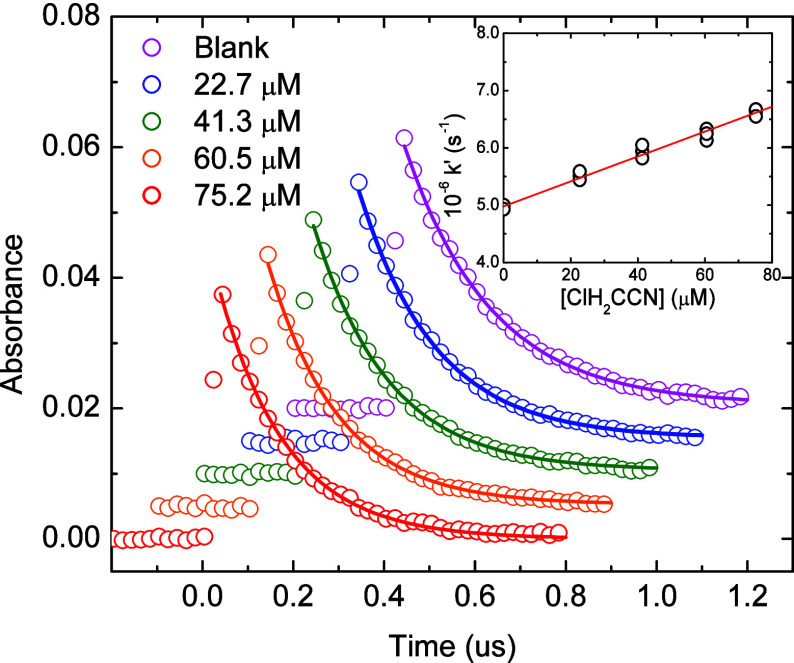
Absorption decay of pulse radiolysis generated hydrated electrons at 720 nm in 0.5 M *tert*-butanol in the presence of different concentrations of chloroacetonitrile (CAN). Decay kinetics offset in time and intensity to aid clarity. Solid lines are fitted exponential decays, corresponding to pseudo-first-order rate constants of (4.95 ± 0.06) × 10,^6^ (5.51 ± 0.10) × 10,^6^ (5.94 ± 0.13) × 10,^6^ (6.25 ± 0.08) × 10,^6^ and (6.64 ± 0.15) × 10^6^ s^–1^, for zero (blank), 22.7, 41.3, 60.5, and 75.2 uM added CAN, respectively. Inset: Second-order plot of fitted k’ values against CAN concentration. Solid line corresponds to rate constant of *k*_5_ = (2.45 ± 0.16) × 10^10^ M^–1^ s^–1^.

Several different experimental conditions for the measurement of the hydrated electron rate constant were conducted to ensure that the most accurate data were obtained. It was found that the traditional pulse radiolysis measurement method of continuously sparging the HAN solution with Ar/N_2_ to maintain deaerated conditions gave more irreproducible/scattered rate constants, consistent with significant volatilization of the HANs occurring. Consistent rate constants (see Table 1) were only obtained when these kinetics were measured with pure HAN additions to aerated water.^69^

**Table 1:** Summary of Measured Rate Constants of This Study^a^

**Compound**	**e** ^**–**^_**aq**_ **Rate Constant (M** ^**–1**^ **s** ^**–1**^ **)**	**H** ^**•**^ **Rate Constant (M** ^**–1**^ **s** ^**–1**^ **)**	^**•**^ **OH Rate Constant (M** ^**–1**^ **s** ^**–1**^ **)**	**SO**_**4**_^**–•**^ **Rate Constant(M** ^**–1**^ **s** ^**–1**^ **)**
Chloroacetacetonitrile	**(2.45 ± 0.08) × 10** ^**10**^	**(1.19 ± 0.05) × 10** ^**7**^	**(4.30 ± 0.09) × 10** ^**7**^	**(1.15 ± 0.11) × 10** ^**6**^
Dichloroacetonitrile	**(2.38 ± 0.07) × 10** ^**10**^	**(2.34 ± 0.03) × 10** ^**7**^	**(4.66 ± 0.16) × 10** ^**7**^	**(1.03 ± 0.29) × 10** ^**6**^
Trichloroacetonitrile	**(1.03 ± 0.02) × 10** ^**10**^	**(3.53 ± 0.17) × 10** ^**7**^	**(5.50 ± 0.19) × 10** ^**6**^	**∼ 2 × 10** ^**5**^
Bromoacetonitrile	**(2.70 ± 0.09) × 10** ^**10**^	**(1.58 ± 0.03) × 10** ^**8**^	**(7.02 ± 0.09) × 10** ^**7**^	**(4.21 ± 0.60) × 10** ^**6**^
Dibromoacetonitrile	**(4.85 ± 0.19) × 10** ^**10**^	**(3.97 ± 0.12) × 10** ^**8**^	**(1.00 ± 0.03) × 10** ^**8**^	**(8.09 ± 0.95) × 10** ^**5**^
Acetonitrile	∼4 × 10^7^ M^–1^ s^–1^ ^54^	1.4 × 10^6^ ^55^	2.2 × 10^7^ ^56^	---
Chloromethane	4.6 × 10^8^ M^–1^ s^–1^ ^57^	∼6 × 10^4^ ^55^	---	---
Dichloromethane	6.0 × 10^9^ M^–1^ s^–1^ ^58^	4 × 10^6^ ^55^	5.8 × 10^7^ ^59^	---
Trichloromethane	3.0 × 10^10^ M^–1^ s^–1^ ^60^	1.1 × 10^7^ ^55^	0.5 – 5.0 × 10^7^ ^38^	---
Bromomethane	---	---	---	---
Dibromomethane	2.3 × 10^10^ M^–1^ s^–1^ ^61^	4.1 × 10^8^ ^61^	9.5 × 10^7^ ^62^	---
Methane	---	<10^5^ ^55^	1.2 × 10^8^ ^63^	<10^6^ ^64^
Chloronitromethane	3.0 × 10^10^ M^–1^ s^–1^ ^65^	---	1.94 × 10^8^ ^65^	---
Dichloronitromethane	3.2 × 10^10^ M^–1^ s^–1^ ^65^	---	5.12 × 10^8^ ^65^	---
Trichloronitromethane	2.1 × 10^10^ M^–1^ s^–1^ ^66^	---	4.97 × 10^7^ ^66^	---
Bromonitromethane	3.1 × 10^10^ M^–1^ s^–1^ ^65^	---	8.36 × 10^7^ ^65^	---
Dibromonitromethane	3.0 × 10^10^ M^–1^ s^–1^.^67^	---	4.75 × 10^8^ ^67^	---
Nitromethane	2.2 × 10^10^ M^–1^ s^–1^ ^68^	4.0 × 10^7^ ^55^	---	---

a Values of this study in bold. --- = no reported value.

For these nonsparged measurements, the observed e_aq_^–^ decay kinetics were fitted to an exponential rate law:

$$Abs=Abs^{{}^{\circ}}e^{-k^{\prime }t}$$ 4

where *Abs* is the measured absorption at time t, *Abs°* is the initial intensity and *k’* is the pseudo-first-order decay rate constant fitted. By plotting these *k’* values against the [HAN] the second order rate constant for chloroacetonitrile reaction:

$${e_{\mathrm{aq}}}^{-}+{\mathrm{ClCH}}_{2}\mathrm{CN}\to \mathrm{products}$$ 5

is determined as *k*_5_ = (2.45 ± 0.16) × 10^10^ M^–1^ s^–1^ (see Figure 1, Inset).

As expected, the e_aq_^–^ reduction reaction rate constants for these HANs are fast, approaching the diffusion-controlled limit in water. Only minimal e_aq_^–^ reactivity with acetonitrile itself occurs (*k* = 4 × 10^7^ M^–1^ s^–1^)^54^ indicating that this radical’s reduction of the HANs would result in a halide ion and the corresponding carbon-centered radical. An overall increase in reactivity is seen for more halogen atom substitution, and brominated HAN’s were faster than chlorinated species. These e_aq_^–^ kinetic data can be directly compared to this radical’s reaction with the similarly substituted halomethanes and halonitromethanes (Table 1). For the halomethanes, there are order of magnitude increases for additional chlorine-atom substitution (mono- to trichloromethane) and the reported value of dibromomethane is similar to that of chloroform.^54,57,58,60,61^ No value could be found for hydrated electron reaction with bromomethane in water, and no reduction of methane by the hydrated electron is expected to occur. In contrast, the hydrated electron reduction rate constants for halogenated nitromethanes^65–67^ show fairly consistent values, that are effectively independent of the degree of halogen substitution. This is attributed to the fast reduction of the parent nitromethane^68^ dominating the reactivity. However, the rate constant value for the fully chlorinated species (trichloronitromethane, chloropicrin) was found to be slightly slower (2.1 × 10^10^ M^–1^ s^–1^)^66^ than for mono- and disubstituted species (ca. 3 × 10^10^ M^–1^ s^–1^)^65,67^ which is the same trend as observed here for the HANs of this study. Analogously, the reported rate constant for e_aq_^–^ reduction of carbon tetrachloride (CCl_4_) in water ((1.3 – 2.4) × 10^10^ M^–1^ s^–1^)^38^ is slightly slower than that for chloroform.

### Hydrogen Atom (H^•^) Rate Constants

The reaction of hydrogen atoms with the haloacetonitriles was monitored using the established EPR method. The general expression for the effective damping rate of the FID in these experiments is given by^48–50^

$$\frac{1}{T_{2}\left(eff\right)}=\frac{1}{T_{2}^{o}}+k_{s}\left[S\right]+\sum k_{ex}^{i}\left[R_{i}\right]$$ 6

where *k*_s_[S] is the H^•^ atom scavenging rate and ∑k^i^_ex_[R_i_] represents the spin-dephasing contribution of second-order spin exchange and recombination reactions between H^•^ atoms and other free radicals. At the radiation doses typically used in these experiments the latter term is not negligible for acidic pH’s. This effect has been observed previously as a slight dose (pulse width) dependence of the measured scavenging curves.^70–72^ However, the acidity improves the EPR signals obtained due to the increase in H^•^ atom concentration through the rapid hydrated electron reaction with the proton:^38^

$${e_{\mathrm{aq}}}^{-}+H^{+}\to H^{•},k_{7}=2.1\times 10^{10}\;M^{-1}\;s^{-1}$$ 7

Relatively high acidity (pH 1.0) was used to ensure that the formed hydrated electrons reacted via Reaction 7 rather than with the added HAN. The radiolytically produced ^•^OH radicals were scavenged by the addition of ca. 7.3 mM methanol. Equation[Disp-formula eq8] gives the general reaction of H^•^ atoms with the chloroacetonitriles, andFigure [Fig fig2] shows the measured H^•^ atom reactivity (1/*T*_2_(eff)) with chloroacetonitrile

$$H^{•}+{\mathrm{Cl}}_{x}H_{y}\mathrm{CCN}\to H^{+}+{\mathrm{Cl}}^{-}+{\mathrm{Cl}}_{x-1}H_{y}C^{•}\mathrm{CN},x=1-3,\;y=3-x$$ 8

at the used 12, 25, and 55 ns pulse widths, giving *k*_s_ values (slopes) of (1.28 ± 0.04) × 10,^7^ (1.41 ± 0.04) × 10,^7^ and (1.61 ± 0.06) × 10^7^ M^–1^ s^–1^, respectively, and demonstrating that such spin exchange does slightly affect the measured rate constants for this system. From the average beam currents measured on a shutter positioned before the irradiation cell for these three pulse widths, the weighted, extrapolated, zero-dose rate constant calculated (Figure 2, Inset) was *k*_*7*_ = (1.19 ± 0.05) × 10^7^ M^–1^ s^–1^. The H^•^ atom rate constants for all the haloacetonitriles of this study are summarized in Table 1.

**Figure 2:**
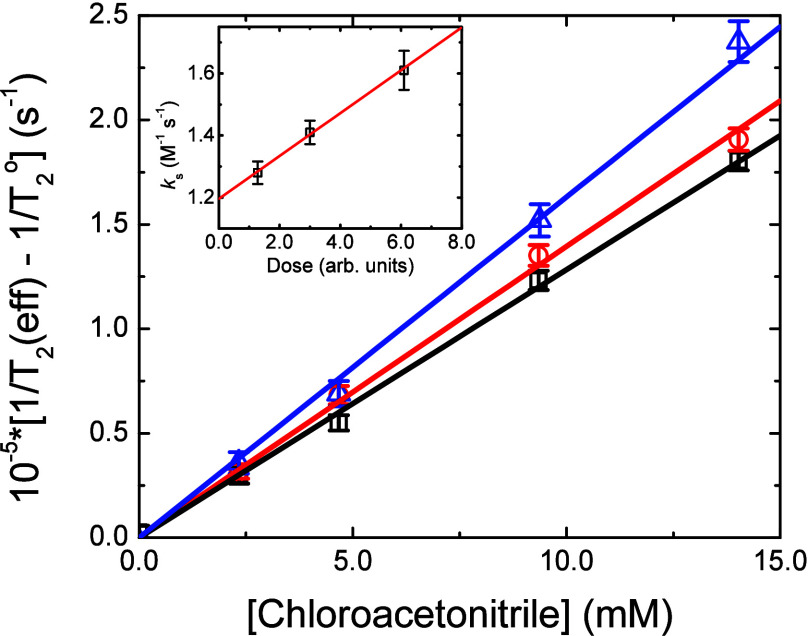
Dose dependence of the aqueous hydrogen atom scavenging rate constant measurements for chloroacetonitrile reaction at pH 1.0 and 21.3 °C using the Van de Graaff 55 ns (**Δ**), 25 ns (**O**), and 12 ns (◘) pulse widths. Solid lines are error-weighted, linear, fits to the intercept-corrected rate constants, corresponding to calculated second-order values of (1.61 ± 0.06) × 10^7^ (1.41 ± 0.04) x 10^7^, and (1.28 ± 0.04) × 10^7^ M^–1^ s^–1^, respectively. **Inset**: Rate constant extrapolation to zero dose for aqueous hydrogen atom reaction with chloroacetonitrile at pH 1.0 and 21.3 °C. Error bars shown correspond to one standard deviation obtained from the linear fit to the FID scavenging plots. Intercept value corresponds to the corrected reaction rate constant of *k*_8_ = (1.19 ± 0.05) × 10^7^ M^–1^ s^–1^.

Free hydrogen atoms in aqueous solutions typically react with saturated organic substrates by a H-atom abstraction, giving H_2_ and carbon centered radicals. However, for these haloacetonitriles, the faster rate constant measured for trichloracetonitrile, (3.53 ± 0.17) × 10^7^ M^–1^ s^–1^, compared to chloroacetonitrile, (1.19 ± 0.05) × 10^7^ M^–1^ s^–1^, dichloroacetonitrile (2.34 ± 0.03) × 10^7^ M^–1^ s^–1^, and especially acetonitrile itself, (1.4 × 10^6^ M^–1^ s^–1^),^55^ suggests that the majority of this reactivity is with the halogen atoms in these molecules (Equation 8). The stepwise increase in H^•^ atom rate constants is consistent with the increasing chlorine atom substitution. Moreover, the order-of-magnitude faster rate constant for dibromoacetonitrile, (3.97 ± 0.12) × 10^8^ M^–1^ s^–1^, as compared to the dichloroacetonitrile again suggests the reduction to form the halide ion dominates. In addition, these H-atom rate constants are about a factor of 1000x slower than for the reduction by the hydrated electron, consistent with previous observations for the reduction of halogenated organics by the two radicals,^38^ and the large increase for brominated moieties is also observed for the corresponding halomethane reactivity with this radical^52,58^ (see Table 1). Unfortunately, no H^•^ reaction rate constants for the halo-substituted halonitromethanes have been reported.

### Hydroxyl Radical (^•^OH) Rate Constants

The strongly oxidizing ^•^OH radical reaction with the haloacetonitriles was measured using N_2_O-saturated solutions, where the radiolytically formed reducing species (e_aq_^–^, H^•^) are rapidly converted to ^•^OH via the reactions:^38,73^

eaq−+N2O(+H2O)→OH•+OH−+N2⁣k9=9.1×109M−1s−1 9

H•+N2O→OH•+N2⁣k10=2×105M−1s−1 10

The direct reaction of ^•^OH radicals with these haloacetonitriles did not show any transient absorption change in the wavelength range 260–800 nm. Thus, these reaction rate constants were established using competition kinetics, utilizing KSCN as a probe.^38^ In the presence of SCN^–^ and, for example, chloroacetonitrile, upon electron irradiation the following competition for ^•^OH occurs:

OH•+ClH2CCN→products⁣k11 11

OH•+SCN−(+SCN−)⇌(SCN)2−•⁣k12=1.05×1010M−1s−1 12

This competition can be analytically solved to give the following expression:

$$\frac{Abs^{{}^{\circ}}}{Abs}=1+\frac{k_{11}\left[ClH_{2}CCN\right]}{k_{12}\left[SCN^{-}\right]}$$ 13

where *Abs*° is the (SCN)_2_^–•^ transient absorption intensity for only KSCN and Abs is the reduced intensity of this absorption when chloroacetonitrile is present. Figure 3 shows the dose-normalized transient absorption kinetics determined for this system in N_2_O-saturated solution, along with the transformed plot according to Equation 13. From this plot, the slope gives the ratio of *k*_11_/*k*_12_, from which a value of *k*_11_ = (4.30 ± 0.09) × 10^7^ M^–1^ s^–1^ was determined. All the haloacetonitrile values of this study are summarized in Table 1.

**Figure 3:**
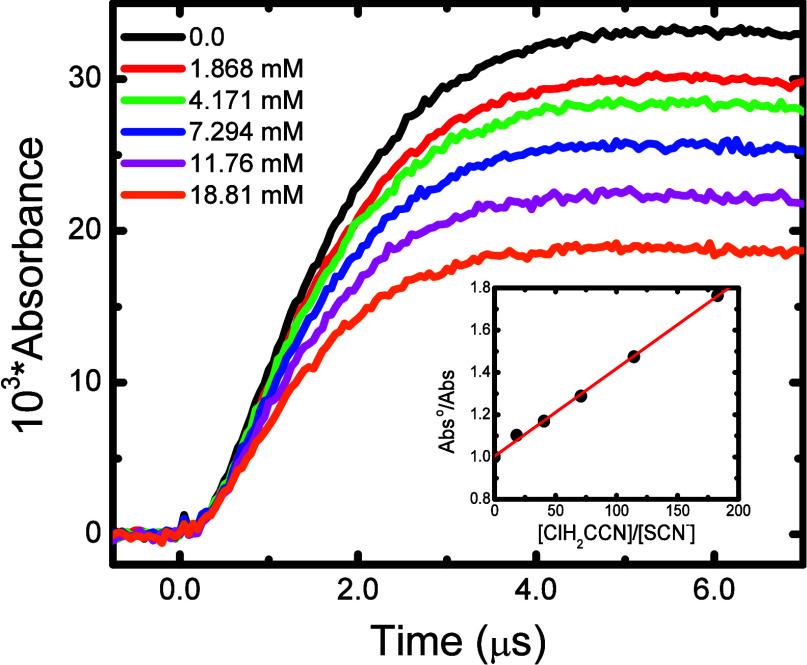
Transient kinetics measured at 475 nm for 102.9 **μ**M KSCN in N_2_O-saturated solution at 23.1 °C in the absence (**—**) and presence of various amounts of ClH_2_CCN: 1.87, 4.17, 7.29, 11.76 and 18.81 mM. Inset: Transformed competition-kinetics plot from peak transient absorbances. Solid line corresponds to weighted linear fit, with intercept 1.009 ± 0.009, and slope of (4.10 ± 0.09) × 10^–3^, R^2^ = 0.995, corresponding to a reaction rate constant of *k*_11_ = (4.30 **±** 0.09) × 10^7^ M^–1^ s^–1^.

The literature rate constants for **^•^** OH reaction with the halomethanes and halonitromethanes (Table 1) show different trends. The values for methylene chloride and chloroform range from (2–5) × 10^7^ M^–1^ s^–1^ although chloroform shows an order of magnitude variation among its reported values.^38^ Dibromomethane was reported to react slightly faster at 9.5 × 10^7^ M^–1^ s^–1^ and no **^•^** OH rate constant for chloromethane in water was found. The reported data for the nitromethanes showed slightly faster reactivity for chloronitromethane (1.9 × 10^8^ M^–1^ s^–1^) and dichloronitromethane (5.1 × 10^8^ M^–1^ s^–1^) while the fully chlorinated species was an order-of-magnitude slower, (5.0 × 10^7^ M^–1^ s^–1^). Bromonitromethane had slightly slower reactivity than chloronitromethane, likely due to the greater steric hindrance of the larger bromine atom in this molecule. In contrast, the dibromonitromethane species rate constant was much faster (4.8 × 10^8^ M^–1^ s^–1^), indicating significant additional activation due to the second Br atom presence.

In this study, the measured **^•^** OH rate constants for HANs showed that these species predominately undergo H-atom abstraction, with dibromoacetonitrile exhibiting the highest reactivity (1.00 × 10^8^ M^–1^ s^–1^). The measured values for chloroacetonitrile (4.30 × 10^7^ M^–1^ s^–1^), dichloroacetonitrile (4.66 × 10^7^ M^–1^ s^–1^), and bromoacetonitrile (7.02 × 10^7^ M^–1^ s^–1^) follow a trend similar to that observed for halonitromethanes, where bromine substitution enhances this radical's reactivity. The increased activation for dibromoacetonitrile aligns with the trend seen in dibromonitromethane, further supporting the role of bromine in accelerating ^•^OH oxidation. The order-of-magnitude slower rate constant for trichloracetonitrile suggests a much slower Cl^–^ ion abstraction reaction is occurring as observed previously for **^•^** OH reaction with chloramines in aqueous solution.^74^

### Sulfate Radical (SO_4_^–•^) Rate Constants

The sulfate radical is another strong oxidant (E^o^ = 2.3 V vs NHE^75^) that can be generated in water in several ways, notably through the reaction of added persulfate (S_2_O_8_^2–^) with reducing e_aq_^–^ or H**^•^** atoms:^38^

$${e_{\mathrm{aq}}}^{-}+S_{2}{O_{8}}^{2-}\to {{\mathrm{SO}}_{4}}^{2-}+{{\mathrm{SO}}_{4}}^{-•},k_{14}=1.2\times 10^{10}\;M^{-1}\;s^{-1}$$ 14

$$H^{•}+S_{2}{O_{8}}^{2-}\to H^{+}+{{\mathrm{SO}}_{4}}^{2-}+{{\mathrm{SO}}_{4}}^{-•},k_{15}=1.4\times 10^{7\;}M^{-1}\;s^{-1}$$ 15

or by photolytically induced cleavage of the S–S bond:^76^

$$S_{2}{O_{8}}^{2-}+\mathrm{hv}\to {{\mathrm{SO}}_{4}}^{-•},\varphi =1.4$$ 16

or by persulfate reaction with a transition metal ion (e.g., Fe^2+^, Ag^+^, Ni^2+^, Sn^2+^):^77^

$$S_{2}{O_{8}}^{2-}+M^{2+}\to M^{3+}+{{\mathrm{SO}}_{4}}^{2-}+{{\mathrm{SO}}_{4}}^{-•}$$ 17

The sulfate radical typically reacts by electron abstraction from electron-rich centers in molecules, or by aliphatic hydrogen atom abstraction:^78,79^

$${{\mathrm{SO}}_{4}}^{-•}+R-H\to {{\mathrm{SO}}_{4}}^{2-}+{\left(R-H\right)}^{+•}$$ 18

$${{\mathrm{SO}}_{4}}^{-•}+R-H\to H^{+}+{{\mathrm{SO}}_{4}}^{2-}+R^{•}$$ 19

This is in contrast to hydroxyl radical-based oxidations that occur mainly by hydrogen atom abstraction and/or addition to aromatic constituents.^38^ The sulfate radical exhibits a strong absorbance at 450 nm,^53^ which allows it to be directly observed using transient UV/visible absorption spectroscopy.

For our electron pulse radiolysis experiments, the sulfate radical was generated from the hydrated electron solution, as above, which also contained 0.10 M K_2_S_2_O_8_. At this high persulfate concentration Reaction 14 dominates, quantitatively converting the hydrated electron to SO_4_^–•^. Figure 4 shows typical decay data obtained for the reaction of this radical with chloroacetonitrile,

$${{\mathrm{SO}}_{4}}^{-•}+{\mathrm{ClH}}_{2}\mathrm{CCN}\to \mathrm{products}$$ 20

**Figure 4:**
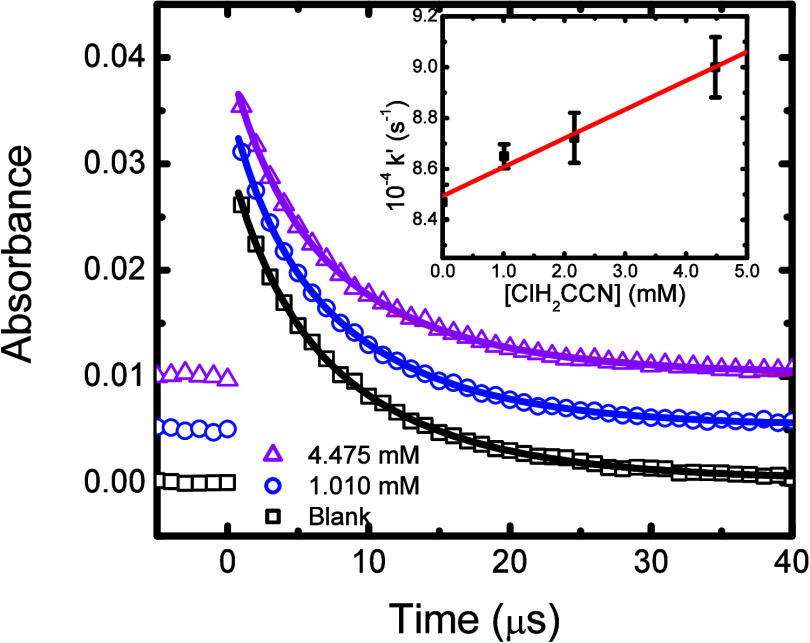
Sulfate radical transient decays observed at 450 nm in aerated 0.10 M K_2_S_2_O_8_ solution containing zero (**◘**), 1.01 (**O**), and 4.48 (**Δ**) mM ClH_2_CCN at 24.2 °C. Fitted lines correspond to mixed-order decays. Data offset to aid visibility. Inset: Second-order reaction rate constant determination for reaction 20 using the averaged fitted pseudo-first-order component data from the individual trace fits. Solid line corresponds to *k*_20_ = (1.15 **±** 0.11) × 10^6^ M^–1^ s^–1^, R^2^ = 0.922.

The slow reactivity observed for this oxidizing radical meant that significant second-order radical combination occurred:^80^

$$2{{\mathrm{SO}}_{4}}^{-•}\to S_{2}{O_{8}}^{2-},,2k=8.8\times 10^{8}\;M^{-1}\;s^{-1}$$ 21

To account for the simultaneous occurrence of both reactions 20 and 21 the observed transient decays were fitted with a mixed-order decay function, whose integrated form is

$$\frac{{\mathrm{Abs}}^{{}^{\circ}}\;*\;k_{20}}{k_{20}\;*\;e^{\left(k_{20}\;*\;t\right)}-2\;*\;{\mathrm{Abs}}^{{}^{\circ}}\;*\;k_{21}\;*\;(1-e^{\left(k_{20}\;*\;t\right)})}+B$$ 22

where *Abs*° is the fitted initial intensity and B is a baseline adjustment parameter. Plotting the pseudo-first-order component of this mixed-order fit against the chloroacetonitrile concentration (see Figure 4, Inset) gives the second-order rate constant for this reaction as *k*_20_ = (1.15 ± 0.11) × 10^6^ M^–1^ s^–1^. All the values obtained for SO_4_^–•^ radical reaction are summarized in Table 1.

Unfortunately, there are no comparative data for SO_4_^–•^ reaction with the halomethanes or the halonitromethanes reported. The measured rate constants for HANs were slow, suggesting that electron abstraction from HANs does not occur; instead, the SO_4_^–•^ radicals react via the less favorable H-atom abstraction process.^81^ For trichloroacetonitrile, the very low estimated rate constant (∼2 × 10^5^ M^–1^ s^–1^) suggests that impurity reactions may also be contributing.

Overall, based on these measured rate constants, the slow oxidation of the haloacetonitriles suggests that using an oxidative-based AO/RP would not be practical for large-scale HAN-contaminated wastewater treatment, with ^•^OH/SO_4_^–•^ side reactions of real-world water matrix components (e.g., bicarbonate/carbonate,^38^ dissolved organic matter,^82–84^ nitrite,^38^ etc.) dominating instead. The much faster reduction rate constants measured for these compounds suggest that using *in situ* generated hydrated electrons could be feasible in real-world waters if this radical’s reaction with dissolved oxygen (ca. 250 μM)^85^

$${e_{\mathrm{aq}}}^{-}+O_{2}\to {O_{2}}^{-•\;},k=1.9\times 10^{10}\;M^{-1}\;s^{-1\;38}$$ 23

^38^ could be overcome. However, for typical haloacetonitrile DBP concentrations in water (ca. 1.8 **μ**g/L,^86^ ∼ 10 nM), a simple relative rates analysis predicts that less than 0.01% of generated hydrated electrons would react with these contaminants (assuming that the other water matrix components did not significantly react). This low reduction-based degradation efficiency could be significantly enhanced by the product superoxide anion radical (O_2_^–**•**^) also reacting with HANs, but no rate constants for these species have been reported. There is one reported value for O_2_^–**•**^ reaction with chloroform (k = 2.3 × 10^8^ M^–1^ s^–1^)^87^ but a preliminary measurement in this study for trichloroacetonitrile showed only minimal superoxide reactivity (Figure 5) with an estimated rate constant of only *k* ≤ 10^6^ M^–1^ s^–1^.

**Figure 5:**
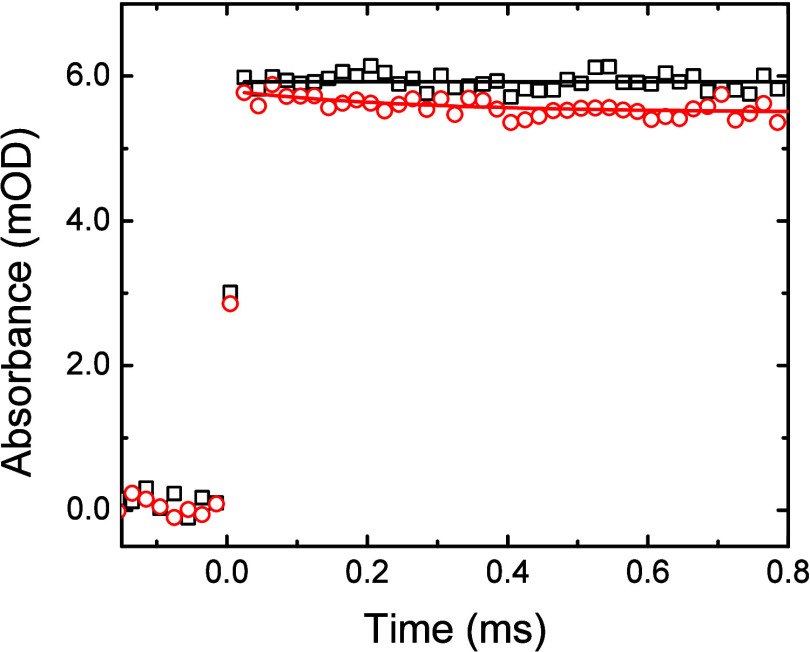
Decay of superoxide radical anion (O_2_^–**•**^) at 280 nm for O_2_-saturated, 0.10 M NaHCO_2_, aqueous solution at pH 7.80 and 22.0 °C; blank (**◘**) and 2.03 mM ClH_2_CCN (**O**). Solid lines correspond to fitted exponential decays, with k’ ∼ 120 ± 300 s^–1^ and k’ = 3800 ± 1300 s^–1^, respectively.

## Conclusions

Absolute rate constants for the reactions of the oxidizing ^•^OH and SO_4_^–•^ radicals, as well as the reducing e_aq_^–^ and H^•^ atom with mono-, di- and tri- chloroacetonitriles and mono- and dibromoacetonitriles have been determined. Significant reduction occurred by the e_aq_^–^ reaction (k = (1–5) × 10^10^ M^–1^ s^–1^), with faster reactivity for the brominated contaminants. Slower but consistent reactivity was seen for H^•^ atom reactions (k = (1 – 40) × 10^7^ M^–1^ s^–1^). Based on structure–activity comparisons both radical-induced HAN reductions are believed to result in halide ions and carbon-centered radicals being produced. This chemistry would augment any direct UV-based AO/RP photodegradation of HANs, which would be expected to produce halogen atoms and carbon-centered radicals in water.^88^ In contrast, only minimal HAN oxidation by ^•^OH (k = (0.4–10) × 10^7^ M^–1^ s^–1^) and SO_4_^–•^ (k = (0.2–4) × 10^6^ M^–1^ s^–1^) occurred. These reactions were attributed to predominant H-atom abstraction from the HAN occurring, also resulting in a carbon-centered radical. A preliminary measurement for the superoxide radical anion (O_2_^–•^) with trichloroacetonitrile showed only minimal reactivity (*k* ≤ 10^6^ M^–1^ s^–1^), indicating that this pathway will not occur to any significant extent. Based on all these kinetic data it is seen that AO/RP treatment of HAN contaminated waters would only be feasible for nonaerated systems, either through direct oxygen removal by sparging an inert gas or by use of sulfite/UV systems.
